# Azobenzene Modified Imidacloprid Derivatives as Photoswitchable Insecticides: Steering Molecular Activity in a Controllable Manner

**DOI:** 10.1038/srep13962

**Published:** 2015-10-05

**Authors:** Zhiping Xu, Lina Shi, Danping Jiang, Jiagao Cheng, Xusheng Shao, Zhong Li

**Affiliations:** 1 Shanghai Key Laboratory of Chemical Biology, School of Pharmacy, East China University of Science and Technology, Shanghai, 200237, China; 2 Shanghai Collaborative Innovation Center for Biomanufacturing Technology, 130 Meilong Road, Shanghai 200237, China

## Abstract

Incorporating the photoisomerizable azobenzene into imidacloprid produced a photoswitchable insecticidal molecule as the first neonicotinoid example of remote control insecticide performance with spatiotemporal resolution. The designed photoswitchable insecticides showed distinguishable activity against Musca both *in vivo* and *in vitro* upon irradiation. Molecular docking study further suggested the binding difference of the two photoisomers. The generation of these photomediated insecticides provides novel insight into the insecticidal activity facilitating further investigation on the functions of insect nicotinic acetylcholine receptors and opens a novel way to control and study insect behavior on insecticide poisoning using light.

Insecticides are widely used in agriculture, industry, horticulture and human buildings, guaranteeing productivity abundance as well as making our lives easier and more enjoyable^1^,^2^. They are designed to regulate insect behaviors causing them death, dysfunction or moving away^3^,^4^. Normally, the activity of an insecticide cannot be controlled intentionally once administrated, making it difficult to elucidate the location and timing of the activity. This uncontrollable property leads to many undesirable secondary effects after application, such as occurrence of resistance^5^, toxic to beneficial wildlife^6^,^7^, food and environmental contamination^8^,^9^. Thus, how to precisely manipulate the activity of an insecticide molecule is a subject of considerable importance for its safe application. The spatiotemporal control of activity will contribute to further understandings of pesticide performance, action mechanism and toxicology profiles and provide better pesticide-environment interactions.

The spatiotemporal regulation of chemical^10^ or biological events^11^,^12^ using artificial molecular photoswitches has been successfully achieved in many systems such as catalyst^13^,^14^,^15^, peptides^16^,^17^, cells^18^, receptors^19^,^20^,^21^,^22^,^23^, ion channel^24^,^25^,^26^,^27^,^28^, nucleic acid^29^ and in living organism^30^,^31^,^32^. Recently, this methodology was applied to the pharmaceutical chemistry generating the photo-controllable molecules which can be activated or deactivated upon irradiation^33^,^34^,^35^,^36^,^37^,^38^. Such photo-responsive bioactive molecules have the merits such as antiresistance, high specificity and low side effect^39^. The efforts in this direction led to a new terminology called photopharmacology^39^.

One elegant way to address the above regulation is to blend a photoisomerizable moiety and a pharmacophore together. The most commonly-used examples take advantage of azobenzene (AB) which can be switched between extended *trans* form and compact *cis* one by irradiation of different wavelength of light. Ilumination with ultraviolet (UV) at wavelength (around 360 nm) leads to *trans*-to-*cis* isomerization, and visible light irradiation switches it back^40^. The two isomers differ greatly in geometry, dipole moment and end-to-end distance and the isomerization occurs with high quantum yields for both transitions^40^,^41^.

However, in the pesticide arena, this technique has never been elaborate for configurational manipulation of pesticide properties. Although the sunlight activation of oxime ether pyrethroids was observed previously, the process is not utilized for further study^42^. As an entry to seeking photoswitchable insecticide, the present investigation describes the azobenzene-modified imidacloprid (AMI) analogues, which enable rapid regulation of activity by light and may lead to the development of novel insecticides driven by easily available physical stimuli.

## Results and Discussion

### Moelcular Design

**Figure 1:**
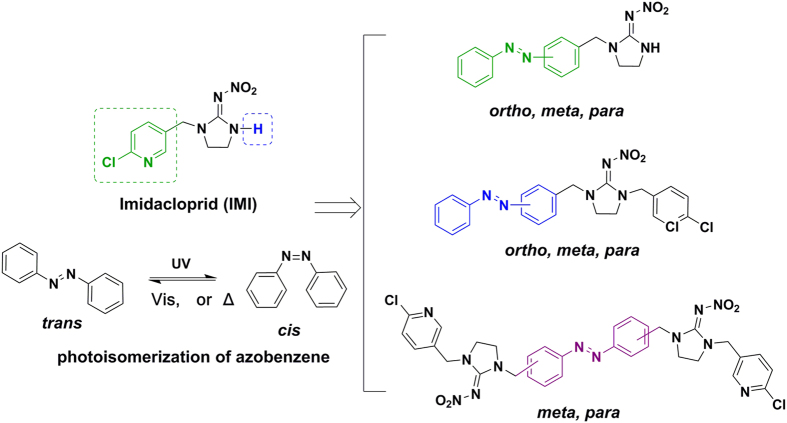
Photoswitchable insecticide design for optical control of activity.

When an active molecule is structurally coupled with the AB, its ability to interact with the target protein might be interfered with by *trans*-*cis* isomerization^39^. Thus, the rationale behind our molecular design involved the fusion of AB to an insecticidal molecule. The design of AMIs was shown in Fig. 1. Our photoswitchable insecticides stemmed from the currently major insecticide, imidacloprid (IMI)^43^. IMI has outstanding efficacy to piercing-sucking insects as an selective insect nicotinic acetylcholine receptor (nAChRs) agonist^44^. It is widely used as a parent compound for neonicotinoids discovery due to the maintenance of potency upon a variety of chemical modifications^45^. Three approaches, replacing the chloropyridinyl with AB, introducing AB into imidazolidine and combining two IMI molecules together, were adopted here for generating the AMIs hoping that the mediation of insecticidal activity with light would be addressed by the introduction of photoswitchable AB.

### Synthesis

**Figure 2:**
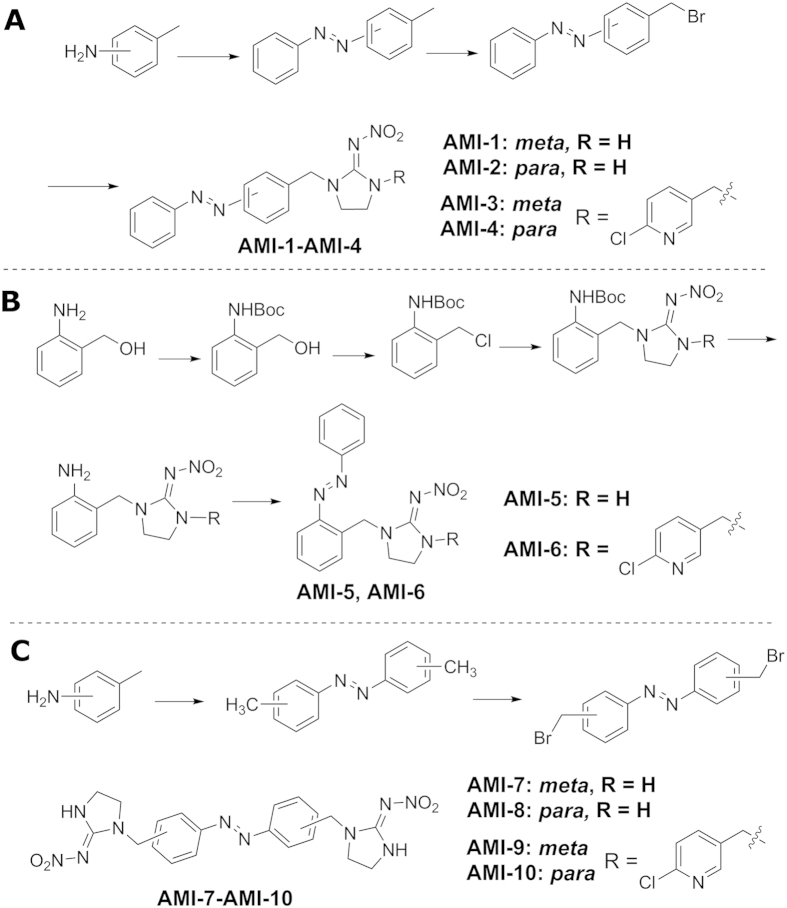
Synthetic route for AMI-1–AMI-10.

Our photoswitchable chemicals consisted of two primary structural features: an AB motif and an IMI pharmacophore. Generally, six main methods are usually employed for constructing azobenzene framework: oxidation of aromatic amines, reduction of nitro compounds, coupling of arylamines with nitroso compounds, diazo-coupling via diazonium, oxidation of hydrazo derivatives and reduction of azoxybenzene^41^. According to the structure features of the target compounds, different multistep syntheses were used. Monovalent **AMI-1 **~ **AMI-4** were prepared starting from m/p-methylaniline via sequential Mills reaction, bromination with NBS and final coupling with IMI analogues (Fig. 2A). During radical bromination, the reaction time and the reactant ratio must be strictly controlled; otherwise unfavorable dibromo- and tribromo-byproducts would form leading to the difficulty of purification. Unfortunately, attempts to synthesize the **AMI-5** and **AMI-6** using a similar method were unsuccessful because the cyclic 2-phenyl-2H-indazole would generate during bromination instead of the monobromide product. Thus, we adopted an alternative route which featured introducing IMI analogues first and AB last (Fig. 2B). Divalent **AMI-7 **~ **AMI-10** were obained by a three-step method. Symmetric azobenzenes were prepared by a modified method via oxidation of m/p-toluidine and radical bromination. Then IMI analogues were fused to them affording the target compounds (Fig. 2C).

### Photoisomerization

**Figure 3:**
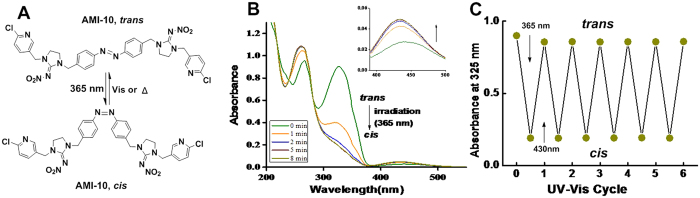
Photochemical properties of AMI-10. (**A**) Trans and cis geometrical structure and isomerization process of AMI-10. (**B**) UV-Vis absorbance of AMI-10 under UV irradiation at 365 nm at varying reaction times (1–8 min). (**C**) Cycling of the conversion of AMI-10 by irradiation alternately with UV and visible light (430 nm).

The photoswitchable behaviors of AMIs at room temperature were characterized by UV/Vis absorption, NMR spectroscopy and HPLC studies. A solution of **AMI-10** in DMSO exhibited a typical absorption spectrum for AB derivatives. Upon irradiation with ultraviolet (365 nm), a gradual intensity decrease at 327 nm and increase at 410–500 nm was detected corresponding to the π–π* and n–π* transition band, respectively (Fig. 3B). The reverse spectral changes were observed upon irradiation at 436 nm (data not shown). All the other compounds gave the similar spectral changes when illuminated with light. The proportions of E/Z isomer at the photostationary states before and after irradiation were determined by HPLC analysis (Table 1). Most of the compounds could produced more than 80% *cis* isomers upon irradiation. No photo bleaching or photo-oxidation could be observed after multiple cycles of photoisomerization or constant irradiation at 365 nm for 1 h (Fig. 3C). Thermal *cis*-*trans* isomerization half-lives of AMI are presented in Table 1. All the compounds had slow thermal isomerization at 25 °C (t_1/2 _> 100 h) indicating that the *cis* isomer was stable in the dark for the duration of biological process. The photoisomerization of **AMI-10** was further identified by ^1^H NMR spectroscopy with the notable appearance of two double peaks at δ 7.25 and 6.85, respectively (Supplementary Fig. S1 online).

### Insecticidal Activity *in Vivo*

The ability of azobenzene to regulate insecticidal activities remains unexplored. To better understand the activity changes induced by light, the efficacy before and after irradiation was tested by leaf-dip method for cowpea aphid (*Aphis craccivora*) and intrathoracic injection against house fly (*Musca domestica*)^46^,^47^. Against cowpea aphid, all the isomers had low activity and no significant difference was observed between two photo isomers (Supplementary Table S1 online). We attribute this observation to the poor solubility at the testing concentrations.

Thus, injection of chemicals into house fly was employed to evaluate their intrinsic activity (Table 2). House fly is a most commonly used indicator for both *in vitro* and *in vivo* insecticidal activity^48^. Replacement of chloropyridinyl in IMI with AB leads to a deleterious effect on *in vivo* activity to house fly as exemplified by analogues **AMI-1**, **2** and **5** with LD_50_ values of more than 10 *μ*g/g irrespective of irradiation or not. **AMI-3** and **4** with AB attachment to IMI at *para*- and *meta*- position, respectively, showed enhanced activity and a slight difference was also realized before and after irradiation. This activity improvement also proved the importance of chloropyridinyl substituent in activity which has been well addressed before^43^. The divalent compounds **AMI-7**–**AMI-10** showed relatively higher insecticidal potency. **AMI-7** and **AMI-8** exhibited increased and decreased activity, respectively, upon irradiation. No obvious difference was observed between the *trans* and *cis* form of **AMI-9**. **AMI-10** was the most effective compound with LD_50_ value of 2.8 *μ*g/g before irradiation and 0.5 *μ*g/g after irradiation, the difference being 5-fold, indicating that the activity can be reversibly adjusted by light. Azobenzene was previously used as an insecticide, but no significant poisoning sign was detected here under the tested concentrations.

### nAChR Binding *in Vitro*

Tritium labeled nitromethylene analogue of IMI ([^3^H]NMI) is a unique reporter for monitoring nAChR binding not only *in vitro* but also *in vivo* due to its extremely high binding potency^48^. Here, the *in vitro* interaction of AMIs with house fly brain nAChR was measured by competitive inhibition of [^3^H]NMI binding (Table 2). Photoisomers of **AMI-1**, **2**, **5**, and **6** had very poor ability to displacing [^3^H]NMI binding (IC_50 _> 2000 nM) leading to difficulty for difference judgement. Slight differences were observed for **AMI-3** and **8** between two isomers, although the activity was very low. A 4-fold difference was achieved on **AMI-4** and **10** upon photoirradiation. Compounds **AMI-9** or **AMI-10** have some similarities with the bis-IMI derivatives disclosed by Kagabu *et al.*^49^,^50^,^51^. Such bis-IMI’s activity was significantly influenced by the spacer types and distance. Among various spacers investigated, the heptamethylene or furan-2,5-dimethylene was the optimal bridge^49^,^50^,^51^. The N-PhN=NPh-N lengths for *trans* and *cis* isomers of **AMI-9** or **AMI-10** are about 12.29 Ǻ and 6.82 Ǻ or 12.59 Ǻ and 8.14 Ǻ, respectively. While for the bis-IMI linked by heptamethylene, the distance is 9.96 Ǻ, close to that of *cis*-**AMI-10**. The shorter or longer distance of the linker may decrease the activity. Furthermore, the lack of heteroatom in the AB linker which is important for stabilizing the interaction with the receptor further attenuated the activity^50^.

### nAChR Docking

**Figure 4:**
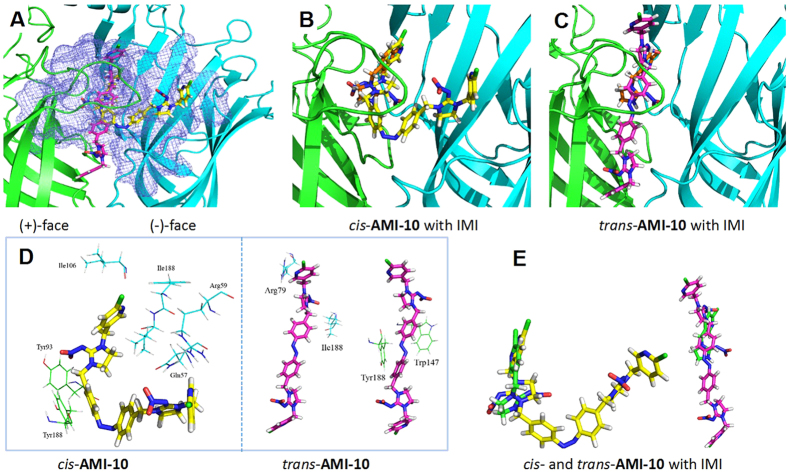
Proposed binding site interactions of AMI-10 with the Aplysia californica acetylcholine binding protein (protein data bank code 2WNJ). (**A**) trans- and cis-AMI-10 nestled in the interfacial binding pocket between the (+)-face (primary, green) and (−)-face (complementary, cyan) subunits with the display of binding region (blue grid). Overlay of the docked structures of cis-AMI-10 with IMI (**B**) and trans-AMI-10 with IMI (**C**), respectively, in the binding pocket. (**D**) Interactions of trans- and cis-AMI-10 with the relevant amino acid residues from the binding pocket. (**E**) Superimpositions of trans- and cis-AMI-10 with IMI in the binding pocket.

In an effort to understand the structure-activity difference in the receptor level, computational modelling of the most active compounds **AMI-10** in its *trans* and *cis* form (Fig. 4A) was carried out using the insect nAChR surrogate *Aplysia* AChBP (protein data bank code 2WNJ)^50^,^52^. The reason we chose 2WNJ here is its ability for adopting the large ligand, which has been verified by Kagabu *et al.* for docking the larger divalent IMI analogues^50^. IMI and NMI, as comparisons, adopted almost the similar binding mode with the protein, indicating the effectiveness of using [^3^H]NMI as indicator for *in vitro* activity. One of the IMI subunits in *cis*-**AMI-10** was situated at the binding pocket to which individual IMI molecule binds, but the nitro-pharmacophores adopt two different binding oritentions (Fig. 4B). For the *trans* isomers, derivations of binding position were observed in camparison with that of the single IMI (Fig. 4C). In the snapshot (Fig. 4D), the AB section of *cis* and *trans* isomers were found to bind with Tyr188 and Trp147, respectively, through π–π stacking interactions. For the *cis*-form, the bent shape of the molecule made the other IMI segment close to the residues forming the H-bond with Gln57 and Arg59, which consolidated the binding with the receptor. However, in the *trans*-isomer, the other IMI moiety extended out of the binding pocket without any interaction with the protein. This observation helps explain the activity difference upon irradiation.

## Conclusion

**Figure 5:**
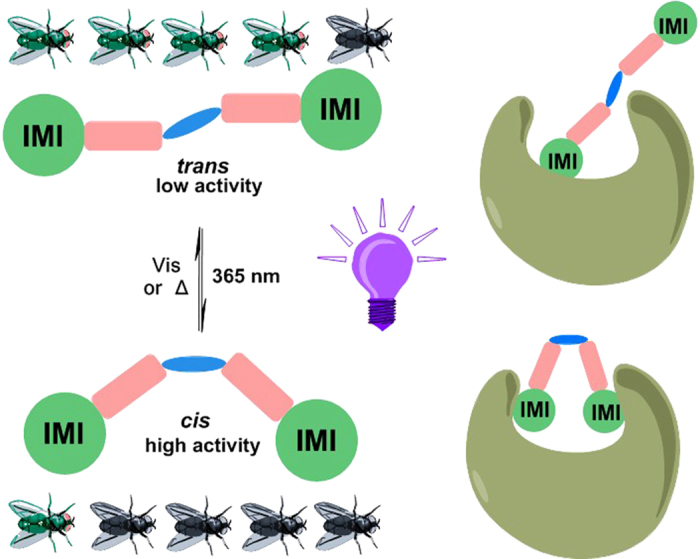
A brief conclusion of our work. The figure was created by ChemBioOffice 2012 and the flies were from the bug template and the lightbulb was created by the shape template and hollow wedged bond.

Using light as external physical stimuli to perturb insecticidal activity was realized by merging IMI with azobenzene. Divalent **AMI-10** had highest potency and gave the most and significant change in insecticidal activity upon irradiation (Fig. 5). Although the complete switching of (on and off) the activity was not achieved, the present findings contribute to developing photoresponsive insecticides. The work illustrates the possibility for modulating insecticide performance using light and for designing conceptually new pesticides.

## Methods

### Synthesis photoswitchable AMIs

The instruments, chmicals, general sythetic procedures and the structural characterization were provided in supplmentary files.

### Photoswitching Experiments

Varian Cary 100 UV-vis spectrophotometer was used for obtaining absorption (UV-Vis) spectra. The solutions of compounds were irradiated with a UV lamp (365 nm). The *cis* to *trans* photoisomeriation were performed with a light-emitting diode (blue light, 450 nm). The corresponding UV-Vis spectrums were collected in supplmentary files. The photostationary states were determined by high performance liquid chromatography (HPLC) measurements and the *trans*/*cis* ratios were calculated at the isosbestic points.

### Insecticidal Activity Assay

[^3^H]NMI (60 Ci/mmol, 98% radiochemical purity) were synthesized by Shanghai Ruxu Radiochemicals Inc. (Shanghai, China).

#### Insecticidal activity for cowpea aphids (Aphis craccivora)

The plant leaves of horsebean with about 50 apterous adults were dipped in corresponding **AMI**s solutions containing Triton X-100 (0.1 mg L^−1^) for 5 s and the excess solutions were sucked out with filter paper. Then the burgeons were positioned in the conditioned room (25 ± 1 °C). Water with Triton X-100 (0.1 mg L^−1^) was used as control. Twenty-four hour after treatment, the mortality rates were counted. Each treatment had three repetitions and the data were subjected to probit analysis.

#### Insecticidal test for house fly (Musca domestica)

House fly adults were anesthetized with carbon dioxide for 10 min and then were treated with the **AMI**s dissolved in distilled water or 10% DMSO aqueous solution by intrathoracic injection (0.4 *μ*L for each fly). Twenty flies were treated for each dosage with duplicate samples. The mortality rates were counted twenty-four hours after treatment. Each treatment had three repetitions and the data subjected to probit analysis.

### nAChR binding assays *in vitro*

House fly heads were collected after the fly adults frozen with liquid nitrogen were broken into body parts. Then the heads were homogenized in 0.1 m*M* EDTA, 0.32 *M* sucrose and 100 m*M* sodium phosphate (pH 7.4). The homogenate was treated by filtering through four layers of 64-*μ*m mesh nylon screen. After centrifuging at 500 g for 30 min, the supernatant was collected and again filtered through using the method above. After centrifuging at 25,000 g for 60 min at 4 °C, the pellet was resuspended in binding buffer (mixture of 50 m*M* sodium chloride and 10 m*M* sodium phosphate, pH 7.4). These brain membranes were used fresh or stored for up to several weeks at −80 °C. Then 1 n*M* [^3^H]NMI, varying concentrations of neonicotinoids and 300–500 *μ*g membrane protein in a final volume of 500 *μ*L was incubated at 22 °C for 60 min. The reaction mixture was filtered through Whatman GF/B glass-fiber filters prewetted by binding buffer using a Brandel M-24R Harvester (Gaithersburg, MD) to quantitate the bound [^3^H]NMI, followed by two rinses each with binding buffer (5 mL) and final liquid scintillation. Nonspecific binding was determined with unlabeled NMI (20 *μM*). Specific binding was about 80–90% of total binding. The percentage inhibition was determined from two (compounds **AMI-1**, **AMI-2**, **AMI-5**, **AMI-6** and azobenzene whose activities were extremely low) or three separate experiments each with triplicate samples. Half maximal inhibitory concentration (IC_50_) were determined by nonlinear regression analysis for logarithm of inhibitor concentration versus probit percentage inhibition.

### Molecular docking

The most active compounds **AMI-10** in its *trans* and *cis* form and IMI and NMI as the camparisons were docked to *Aplysia californica* acetylcholine binding protein (AChBP) crystal structure 2WNJ chain A/E interface using GOLD v3.2 software (The Cambridge Crystallographic Data Centre, Cambridge, U.K.). The Goldscore scoring function was applied to evaluate the docking results. The 2WNJ can bound with a large ligand and was usually used as a prototype for investigating the binding of neonicotinoids. The ligands were drawn and optimized by Maestro 9.0 (Schrödinger, LLC, 2013). All atoms within 10 Å around ZY7 in 2WNJ were selected as binding pocket and used in the docking study. The Goldscore values of the **AMI-10** in its *trans* and *cis* form were 38.16 and 80.27, respectively.

## Additional Information

**How to cite this article**: Xu, Z. *et al.* Azobenzene Modified Imidacloprid Derivatives as Photoswitchable Insecticides: Steering Molecular Activity in a Controllable Manner. *Sci. Rep.* **5**, 13962; doi: 10.1038/srep13962 (2015).

## Supplementary Material

Supplementary Information

## Figures and Tables

**Table 1:** Maximum absorbance wavelength (λ_max_, nm), ratio of *trans* and *cis* isomers and the rate of thermal relaxation of AMIs.

Compd.	*λ*_max_π-π* (*trans*, nm)	nonirradiated *trans*:***cis***	*λ*_max_ n-π* (*cis*, nm)	irradiated *trans*:*cis*	t_1/2_(h)
**AMI-1**	318	92:8	429	16:84	1386.3
**AMI-2**	321	97:3	432	14:86	341.3
**AMI-3**	317	93:7	431	13:87	990.2
**AMI-4**	322	94:6	433	15:85	348.4
**AMI-5**	320	94:6	436	12:88	128.3
**AMI-6**	322	92:8	433	12:88	152.4
**AMI-7**	321	98:2	432	14:86	198.5
**AMI-8**	326	99:1	434	16:84	137.0
**AMI-9**	319	96:4	429	15:85	107.3
**AMI-10**	325	95:5	434	16:84	164.3

**Table 2:** Insecticidal activity of AMIs against house fly (*Musca domestica*) and binding affinity (IC_50_) to house fly nAChR.

Compd.	*Musca domestica in vivo*(LD_50_, *μ*g/g)		*Musca domestica in vitro* (IC_50_, [^3^H] NMI, nM)	
	nonirradiated	irradiated	nonirradiated	irradiated
**AMI-1**	>10	>10	>2000	>2000
**AMI-2**	>10	>10	>2000	>2000
**AMI-3**	3.9 ± 0.5	4.4 ± 0.7	250 ± 22	1141 ±106
**AMI-4**	6.5 ± 1.0	9.2 ±1.6	818 ± 92	1219 ± 98
**AMI-5**	>10	>10	>2000	>2000
**AMI-6**	>10	>10	>2000	>2000
**AMI-7**	7.9 ± 1.1	5.3 ± 1.0	1865 ± 151	1726 ± 135
**AMI-8**	6.4 ± 1.3	9.3 ± 1.5	850 ± 76	>2000
**AMI-9**	5.1 ± 0.8	3.0 ± 0.6	1159 ± 141	1337 ± 154
**AMI-10**	2.8 ± 0.4	0.5 ± 0.08	466 ± 55	112 ± 13
**azobenzene**	>10	>10	>2000	>2000
**IMI**	0.05 ± 0.01		28.8 ± 4.0	
