# Supplementary material for: Azobenzene Modified Imidacloprid Derivatives as Photoswitchable Insecticides: Steering Molecular Activity in a Controllable Manner
Source: Sci Rep. 2015 Oct 5;5:13962. doi: 10.1038/srep13962 (PMC4593031; doi:10.1038/srep13962)
Supplement: Supplementary Information [file srep13962-s1.pdf]

Azobenzene Modified Imidacloprid Derivatives as Photoswitchable Insecticides: Steering Molecular Activity in a Controllable Manner

Zhiping Xu[1], Lina Shi[1], Danping Jiang[1], Jiagao Cheng[1], Xusheng Shao[1]*, Zhong Li[1,2]

[1]Shanghai Key Laboratory of Chemical Biology, School of Pharmacy, East China University of Science and Technology, Shanghai, 200237, China

[2]Shanghai Collaborative Innovation Center for Biomanufacturing Technology, 130 Meilong Road, Shanghai 200237, China

Table of contents.

1. Synthesis

2. Photochromism

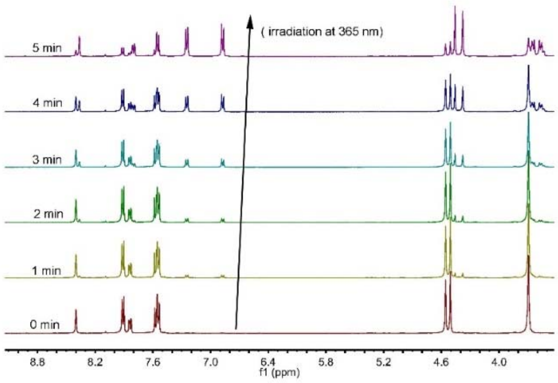

Supplementary Fig. S1. ^{1}H NMR spectral changes of **AMI-10** in DMSO-d_6 upon irradiation with UV light at 365nm.

Supplementary Table S1. Insecticidal activity of **AMIs** against cowpea aphis (*Aphis craccivora*).

**Compd.**	*Aphis craccivora* (in *vivo*) (Mortality, 200 mg L^{-1}, %)	
	nonirradiated	irradiated
**AMI-1**	0	0
**AMI-2**	11.7	0
**AMI-3**	57.7	42.4
**AMI-4**	59.9	53.5
**AMI-5**	21.7	0
**AMI-6**	27.5	11.3
**AMI-7**	32.6	15.7
**AMI-8**	20.0	31.6
**AMI-9**	44.1	52.5
**AMI-10**	31.7	45.9
**azobenzene**	0	0
**IMI**	100	

1. Synthesis

Instruments and Chemicals. Melting points (mp) were recorded on Büchi B540 apparatus (Büchi Labortechnik AG, Flawil, Switzerland) and are uncorrected. ^{1}H NMR and ^{13}C NMR spectra were recorded on Bruker AM-400 (400 MHz) spectrometer with $CDCl_3$ or DMSO-d_6 as the solvent and TMS as the internal standard. Chemical shifts are reported in δ (parts per million) values. Electrospray ionization (ESI) mass spectrometry was performed in a HP 1100 LC-MS spectrometer. Analytical thin-layer chromatography (TLC) was carried out on precoated plates (silica gel 60 F254), and spots were visualized with ultraviolet (UV) light. Column chromatography was performed using silica gel (Hailang, Qingdao) 200-300 mesh. Unless otherwise noted, reagents and solvents were used as received from commercial suppliers. Yields were not optimized. All reactions were carried out under a protective atmosphere of drying nitrogen or utilizing a calcium chloride tube.

The general synthetic methods for compounds **AMI-1-AMI-10** are depicted in Figure **S2**.

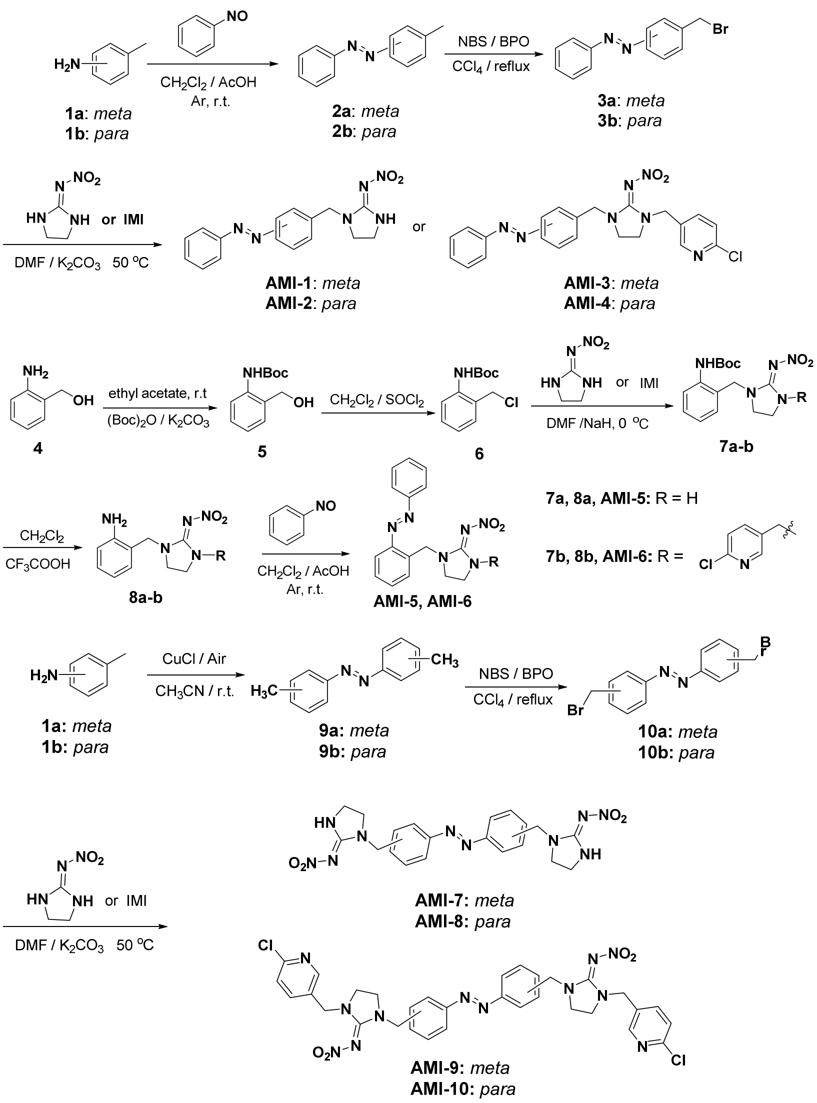

Supplementary Fig. S2. Synthetic routes for compounds **AMI-1-AMI-10**

(E)-1-phenyl-2-(m-tolyl)diazene (***2a***)

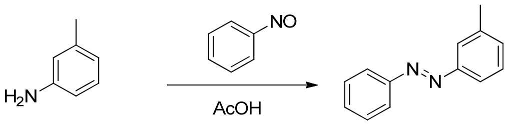

A mixed solution of *m-toluidine* (0.865 g, 8.0 mmol), dichloromethane (12 mL) and acetic acid (18 mL) was stirred in the dark under argon. Then, nitrosobenzene (0.856g, 8.0 mmol) was added slowly with stirring at room temperature. The resulted mixture was stirred for 4 h and the progress of the reaction was monitored by TLC analysis. After completion, saturated aqueous sodium carbonate was added for adjusting the pH values to 7. Then the mixture was diluted with water and extracted with CH_2Cl_2 (20 mL × 4). The combined organic layers were dried over Na_2SO_4 and concentrated. The crude product was further purified by flash column chromatography using petroleum ether and dichloromethane (v:v = 10:1) to offer orange-red oil, which was solidified into orange-red solid after freezing. Yield 88.5 %, mp =

23.0-24.0 °C; ^{1}H NMR (400 MHz, $CDCl_3$): δ 7.95 (dd, J_1 = 1.6 Hz, J_2 = 7.2 Hz, 2H), 7.77 (d, J = 6.0 Hz, 2H), 7.58-7.47 (m, 3H), 7.47-7.41 (m, 1H), 7.33 (d, J = 7.2 Hz, 1H), 2.49 (s, 3H) ppm.

*(E)-1-phenyl-2-(p-tolyl)diazene (**2b**)*

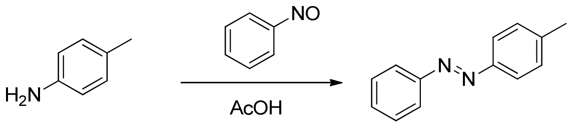

A mixed solution of *p-toluidine* (0.865 g, 8.0 mmol), dichloromethane (12 mL) and acetic acid (18 mL) was stirred in the dark under argon. Then, nitrosobenzene (0.856g, 8.0 mmol) was added slowly with stirring at room temperature. The resulted mixture was stirred for 4 h and the progress of the reaction was monitored by TLC analysis. After completion, saturated aqueous sodium carbonate was added for adjusting the pH values to 7. Then the mixture was diluted with water and extracted with CH_2Cl_2 (20 mL × 4). The combined organic layers were dried over Na_2SO_4 and concentrated. The crude product was further purified by flash column chromatography using petroleum ether dichloromethane (v:v = 10:1) affording the target compounds as orange solid. Yield 92.3 %, mp = 69.8-70.4 °C; ^{1}H NMR (400 MHz, DMSO-d_6): δ 7.88 (dd, J_1 = 1.6 Hz, J_2 = 8.0 Hz, 2H), 7.82 (d, J = 8.0 Hz, 2H), 7.64-7.53 (m, 3H), 7.41 (d, J = 8.0 Hz, 2H), 2.42 (s, 3H) ppm.

*(E)-1-(3-(bromomethyl)phenyl)-2-phenyldiazene (**3a**)*

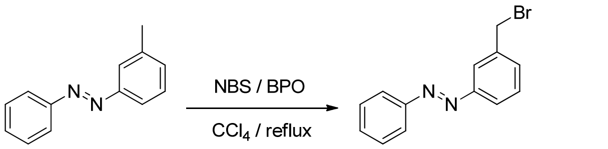

Compound **2a** (1.176 g, 6.0 mmol), bromo-succinimide (NBS, 1.062 g, 6.0 mmol) and benzoyl peroxide (BPO, 0.0726 g, 0.3 mmol) were sequently added into CCl_4 (40 mL). Then, the mixture was stirred under argon at refluxing for 24h and the progress of the reaction was monitored by TLC analysis. After completion, filtrated and washed three times with carbon tetrachloride (5 mL × 4), then the filtrate was evaporated under reduced pressure to remove carbon tetrachloride. Then, the mixture was diluted with water and extracted with CH_2Cl_2 (20 mL × 4). The combined organic layers were dried over Na_2SO_4 and concentrated. The crude product was further purified by column chromatography using petroleum ether and dichloromethane (v:v = 10:1) as eluent affording orange solid. Yield 53.4 %, ^{1}H NMR (400 MHz, $CDCl_3$): δ 8.00-7.93 (m, 3H), 7.93-7.87 (m, 1H), 7.61-7.49 (m, 5H), 4.62 (s, 2H) ppm.

*(E)-1-(4-(bromomethyl)phenyl)-2-phenyldiazene (**3b**)*

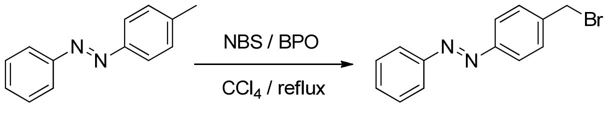

Compound **2b** (1.176 g, 6.0 mmol), bromo-succinimide (NBS, 1.062 g, 6.0 mmol) and benzoyl peroxide (BPO, 0.0726 g, 0.3 mmol) were added sequently into CCl_4 (40 mL). Then, the mixture was stirred under argon at refluxing for 24h and the progress of the reaction was monitored by TLC analysis. After completion, filtrated and washed three times with carbon tetrachloride (5 mL × 4), then the filtrate was evaporated under reduced pressure to remove carbon tetrachloride. Then, the mixture was diluted with water and extracted with CH_2Cl_2 (20 mL × 4). The combined organic layers were dried over Na_2SO_4 and

concentrated. The crude product was further purified by column chromatography using petroleum ether and dichloromethane (v:v = 10:1) as eluent affording orange solid. Yield 75.7 %, mp=112.5-113.5 °C; ^{1}H NMR (400 MHz, $CDCl_3$): δ 7.93-7.86 (m, 4H), 7.55-7.44 (m, 5H), 4.54 (s, 2H) ppm.

*tert-butyl (2-(hydroxymethyl) phenyl) carbamate (**5**)*

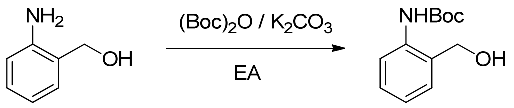

A solution of o-aminobenzyl alcohol (0.984 g, 8.0 mmol), boc-anhydride (8.8 mmol) and K_2CO_3 (1.656 g, 12.0 mmol) was stirred at room temperature for 24h and the progress of the reaction was monitored by TLC analysis. After completion, the reaction mixture was diluted with water and extracted with ethyl acetate (15 mL × 5). The combined organic layers were dried over Na_2SO_4 and concentrated. The crude product was further purified by column chromatography using dichloromethane and ethyl acetate (v:v = 50:1) as eluent. White viscous liquid, yield 93.4 %, ^{1}H NMR (400 MHz, $CDCl_3$): δ 7.89 (d, J = 8.0 Hz, 1H), 7.69 (s, 1H), 7.35-7.26 (m, 1H), 7.16 (d, J = 7.6 Hz, 1H), 7.03 (t, J = 7.6 Hz, 1H), 5.31 (s, 1H), 4.66 (s, 2H), 1.54 (s, 9H) ppm.

*tert-butyl (2-(chloromethyl) phenyl) carbamate (**6**)*

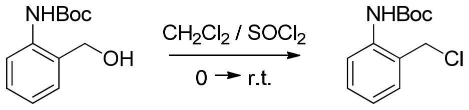

Compound **5** (0.560 g, 2.5 mmol) was dissolved in 10 mL dichloromethane and the mixture was stirred at 0 °C. Then the $SOCl_2$ (0.892 g, 7.5 mmol) in 10 mL dichloromethane was dropwisely added into the reaction mixture. The progress of the reaction was monitored by TLC analysis. After completion, 15% aqueous sodium hydroxide solution was added to adjust the pH value to 7. Then, the reaction mixture was diluted with water and extracted with CH_2Cl_2 (10 mL × 6). The combined organic layers were dried over Na_2SO_4 and concentrated. The crude product was further purified by flash column chromatography using dichloromethane. White solid, yield 87.1 %, mp = 84.4-85.2 °C; ^{1}H NMR (400 MHz, $CDCl_3$): δ 7.88 (d, J = 8.0 Hz, 1H), 7.43-7.34 (m, 1H), 7.33-7.29 (m, 1H), 7.09 (t, J = 7.6 Hz, 1H), 6.78 (s, 1H), 4.64 (s, 2H), 1.56 (s, 9H) ppm.

*(Z)-tert-butyl (2-((2-(nitroimino)imidazolidin-1-yl)methyl)phenyl)carbamate (**7a**)*

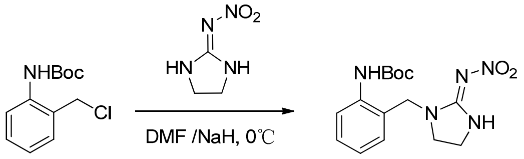

Compound **6** (0.260 g, 2.0 mmol) in 10 mL DMF was stirred at 0°C and NaH (0.482 g, 2.0 mmol) was added in the mixture carefully and keep the mixture stirring for 30 min. Then compound **6** (0.482 g, 2.0 mmol) was added in the mixture. The reaction mixture was kept stirring overnight and the reaction mixture was diluted with water and extracted with dichloromethane CH_2Cl_2 (10 mL × 6). The combined organic layers were dried over Na_2SO_4 and concentrated. The crude product was further purified by column chromatography using dichloromethane and ethyl acetate (v:v = 20:1) as eluent. White solid,

yield 59.3 %, m.p. = 137.9-139.1 °C; ^{1}H NMR (400 MHz, $CDCl_3$): δ 8.12-7.99 (m, 2H), 7.36 (t, J = 7.6 Hz, 1H), 7.15 (d, J = 8.0 Hz, 1H), 7.03 (t, J = 7.6 Hz, 1H), 4.51 (s, 2H), 3.83-3.66 (m, 2H), 3.60-3.48 (m, 2H), 1.53 (s, 9H) ppm.

*tert-butyl (Z)-(2-((3-((6-chloropyridin-3-yl)methyl)-2-(nitroimino)imidazolidin-1-yl)methyl)phenyl)carbamate (**7b**)*

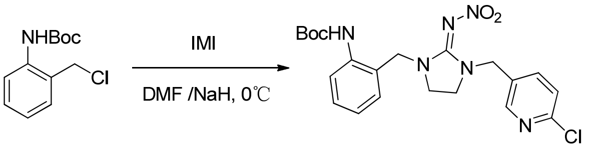

Imidacloprid (0.840 g, 3.3 mmol) in 10 mL DMF was stirred at 0 °C and the NaH (0.2 g, 6.0 mmol) was added in the mixture carefully and was stirring for 30 min. Then compound **6** (0.482 g, 2.0 mmol) was added in the mixture. The reaction mixture was kept stirring overnight and the reaction mixture was diluted with water and extracted with dichloromethane CH_2Cl_2 (10 mL × 6). The combined organic layers were dried over Na_2SO_4 and concentrated. The crude product was further purified by column chromatography using dichloromethane and acetone (v:v = 50:1) as eluent. Light yellow, yield 69.4 %, m.p. = 109.1-109.7 °C; ^{1}H NMR (400 MHz, $CDCl_3$): δ 8.54 (s, 1H), 8.30 (d, J = 2.0 Hz, 1H), 8.03 (d, J = 8.4 Hz, 1H), 7.65 (dd, J_1 = 2.4 Hz, J_2 = 8.0 Hz, 1H), 7.36-7.30 (m, 2H), 7.13 (dd, J_1 = 1.4 Hz, J_2 = 7.5 Hz, 1H), 7.04-6.97 (m, 1H), 4.39 (s, 2H), 4.35 (s, 2H), 3.36-3.27 (m, 2H), 3.25-3.16 (m, 2H), 1.57 (s, 9H) ppm.

*(E)-N-(1-(2-aminobenzyl)imidazolidin-2-ylidene)nitramide (**8a**)*

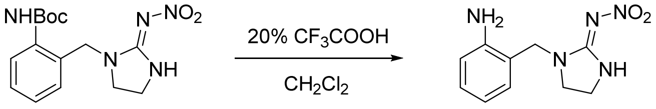

Compound **7a** (1.054 g, 1.5 mmol), trifluoroacetic acid (5 mL) and dichloromethane (20 mL) were stirring for 4h at room temperature. After completion, 15% aqueous sodium hydroxide solution was added to adjust the pH value to 7. The reaction mixture was diluted with water and extracted with dichloromethane (10 mL × 6). The combined organic layers were dried over Na_2SO_4 and concentrated. Light yellow solid, yield 81.6 %, m.p. = 156.0-157.0 °C; ^{1}H NMR (400 MHz, $CDCl_3$): δ 8.06 (s, 1H), 7.17 (t, J = 7.6 Hz, 1H), 7.04 (d, J = 7.6 Hz, 1H), 6.76-6.65 (m, 2H), 4.46 (s, 2H), 4.04 (s, 2H), 3.82-3.71 (m, 2H), 3.58-3.47 (m, 2H) ppm.

*(E)-N-(1-(2-aminobenzyl)-3-((6-chloropyridin-3-yl)methyl) imidazolidin-2-ylidene) nitramide (**8b**)*

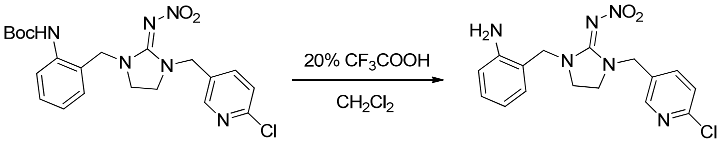

Compound **7b** (0.5 g, 1.5 mmol), trifluoroacetic acid (5 mL) and dichloromethane (20 mL) were stirring for 4h at room temperature. After completion, 15% aqueous sodium hydroxide solution was added to adjust the pH value to 7. The reaction mixture was diluted with water and extracted with dichloromethane (10 mL × 6). The combined organic layers were dried over Na_2SO_4 and concentrated. Light yellow viscous liquid, yield 77.3 %, m.p. = 126.1.0-127.8 °C; ^{1}H NMR (400 MHz, $CDCl_3$): δ 8.30 (s, 1H), 7.63

(dd, *J* = 8.0, 2.0 Hz, 1H), 7.32 (d, *J* = 8.0 Hz, 1H), 7.13 (t, *J* = 7.6 Hz, 1H), 7.02 (d, *J* = 7.6 Hz, 1H), 6.73-6.64 (m, 2H), 4.38 (s, 2H), 4.34 (s, 2H), 3.94 (s, 2H), 3.30-3.23 (m, 2H), 3.23-3.16 (m, 2H) ppm.

*3,3'-dimethyl-azobenzene (**9a**)*

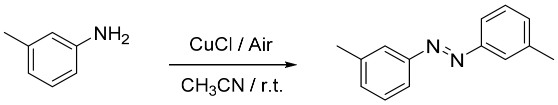

m-toluidine (1.792 g, 16.0 mmol) in acetonitrile (40 mL) was stirred in the dark and then CuCl (0.32 g, 3.2 mmol) was added at room temperature. After stirring overnight, the reaction mixture was diluted with water and extracted with dichloromethane (15 mL × 4). The combined organic layers were dried over Na_2SO_4 and concentrated. The crude product was further purified by flash column chromatography using petroleum ether and dichloromethane (v:v = 10:1) as eluent. Orange solid, yield 61.6 %, ^{1}H NMR (400 MHz, $CDCl_3$): δ 7.69-7.70 (m, 4H), 7.45-7.49 (m, 2H), 7.37 (d, *J* = 8.0 Hz, 2H), 2.42 (s, 6H) ppm.

*4,4'-dimethyl-azobenzene (**9b**)*

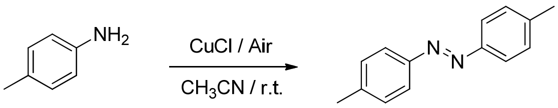

P-toluidine (1.792 g, 16.0 mmol) in acetonitrile (40 mL) was stirred in the dark and then CuCl (0.32 g, 3.2 mmol) was added at room temperature. After stirring overnight, the reaction mixture was diluted with water and extracted with dichloromethane (15 mL × 4). The combined organic layers were dried over Na_2SO_4 and concentrated. The crude product was further purified by flash column chromatography using petroleum ether and dichloromethane (v:v = 10:1) as eluent. Orange solid, yield 84.8 %, m.p. = 143.3-144.1 °C; ^{1}H NMR (400 MHz, $CDCl_3$): δ 7.84 (d, *J* = 8.4 Hz, 4H), 7.34 (d, *J* = 8.0 Hz, 4H), 2.46 (s, 6H) ppm; ^{13}C NMR (100 MHz, $CDCl_3$) δ 150.83, 141.22, 129.72, 122.73, 21.50 ppm.

*(E)-1,2-bis(3-(bromomethyl)phenyl)diazene (**10a**)*

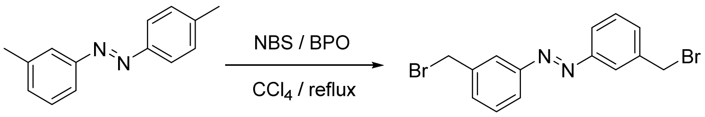

3,3'-dimethyl-azobenzene (0.910 g, 4.3 mmol), bromo-succinimide (NBS, 1.84 g, 10.4 mmol) and benzoyl peroxide (BPO, 0.052 g, 0.215 mmol) were added in CCl_4 (40 mL) and the mixture was stirred under argon at refluxing for 24h and the progress of the reaction was monitored by TLC analysis. After completion, filtrated and washed three times with water affording the orange solid. Yield 61.5 %, m.p. = 140.1-141.6 °C; ^{1}H NMR (400 MHz, $CDCl_3$): δ 7.98 (s, 2H), 7.87 (d, *J* = 8.0 Hz, 2H), 7.66-7.68 (m, 2H), 7.59-7.63 (m, 2H), 4. 85 (s, 4H) ppm.

*(E)-1,2-bis(4-(bromomethyl)phenyl)diazene (**10b**)*

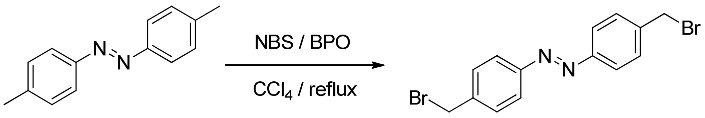

4,4'-dimethyl-azobenzene (0.910 g, 4.3 mmol), bromo-succinimide (NBS, 1.84 g, 10.4 mmol) and benzoyl peroxide (BPO, 0.052 g, 0.215 mmol) were added in CCl_4 (40 mL) and the mixture was stirred under argon at refluxing for 24h and the progress of the reaction was monitored by TLC analysis. After completion, filtrated and washed three times with water affording the orange solid. Yield 52.5 %, m.p. = 196.7-197.5 °C; ^{1}H NMR (400 MHz, $CDCl_3$): δ 7.92 (d, J = 8.4 Hz, 4H), 7.57 (d, J = 8.4 Hz, 4H), 4.58 (s, 4H) ppm.

*(Z)-2-(nitromethylene)-1-(3-((E)-phenyldiazenyl)benzyl)imidazolidine (**AMI-1**)*

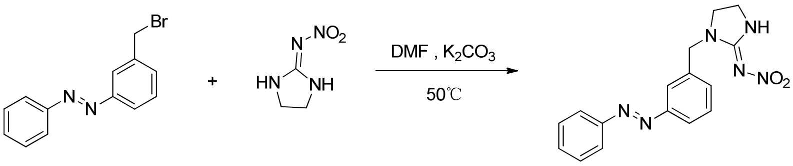

Amino-2-nitromethylene-imidazolidine (0.130 g, 1.0 mmol) and K_2CO_3 (0.166 g, 1.2 mmol) were dissolved in DMF (8 mL) was stirred at 50°C. Then *(E)-1-(3-(bromomethyl)phenyl)-2-phenyldiazene* (1 mmol) in DMF was added in the mixture. Then, the mixture was stirred at 50°C for 24h and the progress of the reaction was monitored by TLC analysis. After completion, the reaction was evaporated under reduced pressure to remove DMF, diluted with water and extracted with dichloromethane (15 mL × 4). The combined organic layers were dried over Na_2SO_4 and concentrated. The crude product was further purified by column chromatography using dichloromethane and acetone (v:v = 15:1) as eluent. Orange solid, 72.3 %, m.p. = 109.8-111.0 °C; ^{1}H NMR (400 MHz, DMSO-d_6): δ 9.03 (s, 1H), 7.96-7.88 (m, 2H), 7.87-7.79 (m, 2H), 7.67-7.55 (m, 4H), 7.50 (d, J = 7.6 Hz, 1H), 4.59 (s, 2H), 3.72-3.61 (m, 2H), 3.57-3.47 (m, 2H) ppm; ^{13}C NMR (100 MHz, DMSO-d_6): δ 160.84, 152.56, 152.32, 138.25, 132.17, 131.21, 130.35, 129.99, 123.08, 122.33, 122.20, 47.74, 45.49, 42.02 ppm; HRMS (ESI): m/z calcd for $C_{16}H_{17}N_6O_2$ $[M+H]^+$ 325.1413, found 325.1421.

*(Z)-2-(nitromethylene)-1-(4-((E)-phenyldiazenyl)benzyl)imidazolidine (**AMI-2**)*

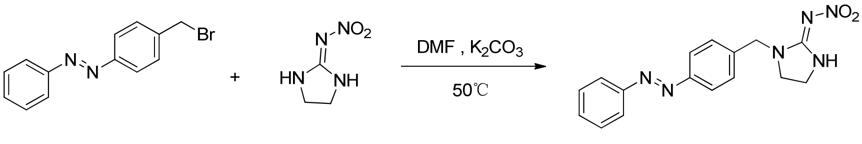

Amino-2-nitromethylene-imidazolidine (0.130 g, 1.0 mmol) and K_2CO_3 (0.166 g, 1.2 mmol) in DMF (8 mL) was stirred at 50°C. Then *E- (4- (bromomethyl)) azobenzene* (1 mmol) in DMF was added in the mixture. The mixture was stirred under 50°C for 24h and the progress of the reaction was monitored by TLC analysis. After completion, the reaction was evaporated under reduced pressure to remove DMF, diluted with water and extracted with dichloromethane (15 mL × 4). The combined organic layers were dried over Na_2SO_4 and concentrated. The crude product was further purified by column chromatography using dichloromethane and acetone (v:v = 15:1) as eluent. Orange solid, yield 65.5 %, m.p. = 193.0-194.3 °C; ^{1}H NMR (400 MHz, DMSO-d_6): δ 9.00 (s, 1H), 8.08-7.79 (m, 4H), 7.66-7.57 (m, 3H), 7.51 (d, J = 8.4 Hz, 2H), 4.56 (s, 2H), 3.73-3.63 (m, 2H), 3.56-3.48 (m, 2H) ppm; ^{13}C NMR (100 MHz, DMSO-

d_6): δ 160.88, 152.41, 151.80, 140.27, 132.03, 129.95, 129.14, 123.35, 123.02, 47.74, 45.54, 42.05 ppm; HRMS (EI): m/z calcd for $C_{16}H_{16}N_6O_2$ [M^+] 324.1335, found 324.1340.

*2-chloro-5-(((E)-2-(nitromethylene)-3-(3-((E)-phenyldiazenyl)benzyl)imidazolidin-1-yl)methyl)pyridine **(AMI-3)***

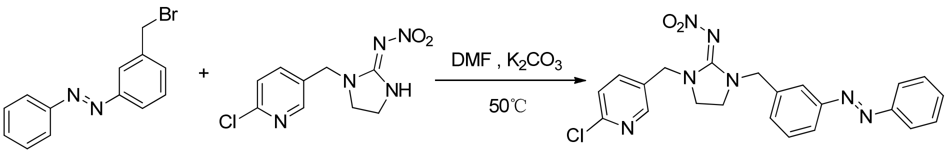

Imidacloprid (0.255 g, 1.0 mmol) and K_2CO_3 (0.138 g, 1.0 mmol) in DMF (10 mL) was stirred at 50°C. Then *(E)-1-(3-(bromomethyl)phenyl)-2-phenyldiazene* (1.0 mmol) in DMF was added. Then, the mixture was stirred at 50°C for 24 h and the progress was monitored by TLC analysis. After completion, the reaction was evaporated under reduced pressure to remove DMF, diluted with water and extracted with dichloromethane extracted with dichloromethane (10 mL × 6). The combined organic layers were dried over Na_2SO_4 and concentrated. The crude product was further purified by column chromatography using dichloromethane and acetone (v:v = 20:1) as eluent. Orange solid, yield 41.0 %, m.p. = 59.1-59.7 °C; ^1H NMR (400 MHz, DMSO-d_6): δ 8.40 (d, J = 2.4 Hz, 1H), 7.98-7.89 (m, 2H), 7.89-7.77 (m, 3H), 7.69-7.58 (m, 4H), 7.54 (d, J = 8.4 Hz, 2H), 4.59 (s, 2H), 4.51 (s, 2H), 3.75-3.65 (m, 4H) ppm; ^{13}C NMR (100 MHz, DMSO-d_6): δ 161.34, 152.61, 152.33, 150.21, 149.92, 139.97, 137.02, 132.19, 131.38, 130.93, 130.35, 129.98, 124.76, 123.08, 122.65, 122.41, 49.63, 47.06, 46.09, 45.84 ppm; HRMS (ESI): m/z calcd for $C_{22}H_{20}N_7O_2{}^{35}Cl$ $[M+Na]^+$ 472.1265, found 472.1264; m/z calcd for $C_{22}H_{20}N_7O_2{}^{37}ClNa$ $[M+Na]^+$ 474.1235, found 474.1240.

*2-chloro-5-(((E)-2-(nitromethylene)-3-(4-((E)-phenyldiazenyl)benzyl)imidazolidin-1-yl)methyl)pyridine **(AMI-4)***

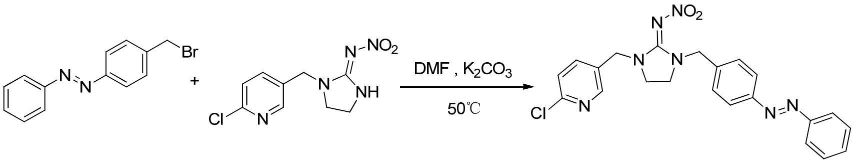

Imidacloprid (0.255 g, 1.0 mmol) and K_2CO_3 (0.166 g, 1.2 mmol) in DMF (10 mL) was stirred at 50°C. Then *(E)-1-(3-(bromomethyl)phenyl)-2-phenyldiazene* (1.0 mmol) in DMF was added. Then, the mixture was stirred at 50°C for 24 h and the progress was monitored by TLC analysis. After completion, the reaction was evaporated under reduced pressure to remove DMF, diluted with water and extracted with dichloromethane extracted with dichloromethane (10 mL × 6). The combined organic layers were dried over Na_2SO_4 and concentrated. The crude product was further purified by column chromatography using dichloromethane and acetone (v:v = 20:1) as eluent. Orange solid, yield 35.4 %, m.p. = 158.8-159.2 °C; ^1H NMR (400 MHz, DMSO-d_6): δ 8.40 (d, J = 2.4 Hz, 1H), 7.94-7.88 (m, 4H), 7.84 (dd, J_1 = 2.4 Hz, J_2 = 8.4 Hz, 1H), 7.65-7.56 (m, 4H), 7.54 (d, J = 8.4 Hz, 2H), 4.56 (s, 2H), 4.51 (s, 2H), 3.73-3.66 (m, 4H) ppm; ^{13}C NMR (100 MHz, DMSO-d_6): δ 161.33, 152.40, 151.97, 150.21, 149.94, 139.98, 139.01, 132.11, 130.93, 129.97, 129.43, 124.79, 123.32, 123.04, 49.63, 47.05, 46.09, 45.91 ppm; HRMS (ESI): m/z calcd

for $C_{22}H_{20}N_7O_2{}^{35}ClNa$ $[M+Na]^+$ 472.1265, found 472.1261; m/z calcd for $C_{22}H_{20}N_7O_2{}^{37}ClNa$ $[M+Na]^+$ 474.1235, found 474.1232.

*(Z)-2-(nitromethylene)-1-(2-((E)-phenyldiazenyl)benzyl)imidazolidine (**AMI-5**)*

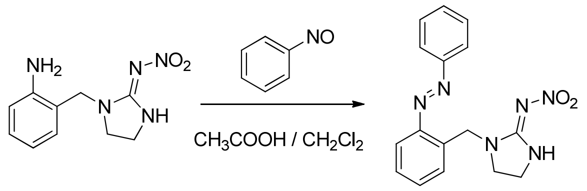

Compound **8a** (0.233 g, 1.0 mmol) in dichloromethane (4 mL) and acetic acid (6 mL) was stirred in the dark under argon at room temperature. Then nitrosobenzene (0.107 g, 1.0 mmol) was added in the mixture. After stirring for 12 h, saturated aqueous sodium carbonate solution was added to adjust the PH value to 7. The reaction mixture was diluted with water and extracted with dichloromethane (10 mL × 6). The combined organic layers were dried over Na_2SO_4 and concentrated. The crude product was further purified by column chromatography using dichloromethane and ethyl acetate (v:v = 30:1) as eluent. Orange solid, yield 52.1 %, m.p. = 136.7-137.4 °C; ^{1}H NMR (400 MHz, DMSO-*d*$_6$): δ 8.94 (s, 1H), 8.05-7.95 (m, 2H), 7.69 (d, J = 8.0 Hz, 1H), 7.65-7.46 (m, 6H), 5.02 (s, 2H), 3.67-3.55 (m, 2H), 3.54-3.43 (m, 2H) ppm; ^{13}C NMR (100 MHz, DMSO-*d*$_6$): δ 160.70, 152.66, 149.85, 135.61, 132.40, 132.24, 130.26, 129.98, 129.24, 123.46, 115.62, 45.52, 44.12, 41.98 ppm; HRMS (ESI): m/z calcd for $C_{16}H_{17}N_6O_2$ $[M+H]^+$ 325.1409, found 325.1421.

*2-chloro-5-(((E)-2-(nitromethylene)-3-(2-((E)-phenyldiazenyl)benzyl)imidazolidin-1-yl)methyl)pyridine (**AMI-6**)*

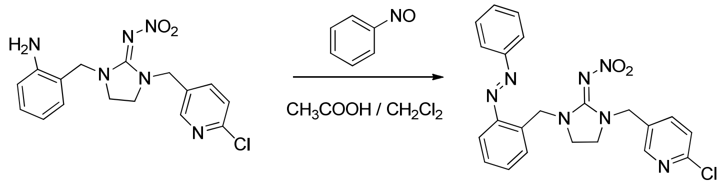

Compound **8b** (0.547 g, 1.52 mmol) in dichloromethane (6 mL) and acetic acid (9 mL) was stirred in the dark under argon at room temperature. Then nitrosobenzene (0.193 g, 1.8 mmol) was added in the mixture. After stirring for 12 h, saturated aqueous sodium carbonate solution was added to adjust the PH value to 7. The reaction mixture was diluted with water and extracted with dichloromethane (15 mL × 6). The combined organic layers were dried over Na_2SO_4 and concentrated. The crude product was further purified by column chromatography using dichloromethane and acetone (v:v = 50:1) as eluent. Orange solid, yield 50.4 %, m.p. = 103.8-104.6 °C; ^{1}H NMR (400 MHz, DMSO-*d*$_6$): δ 8.31 (d, J = 2.4 Hz, 1H), 8.03-7.93 (m, 2H), 7.74-7.50 (m, 7H), 7.50-7.40 (m, 2H), 4.89 (s, 2H), 4.34 (s, 2H), 3.34-3.12 (m, 4H) ppm; ^{13}C NMR (100 MHz, DMSO-*d*$_6$): δ 160.74, 152.68, 150.01, 149.74, 149.61, 139.80, 136.99, 133.36, 132.28, 132.12, 130.34, 129.94, 128.82, 124.64, 123.45, 115.49, 44.76, 43.65, 42.49, 42.37 ppm; HRMS (ESI): m/z calcd for $C_{22}H_{20}N_7O_2{}^{35}ClNa$ $[M+Na]^+$ 472.1267, found 472.1260.

*(E)-1-(3-(((E)-2-(nitromethylene)imidazolidin-1-yl)methyl)phenyl)-2-(3-(((Z)-2-(nitromethylene)imidazolidin-1-yl)methyl)phenyl)diazene (**AMI-7**)*

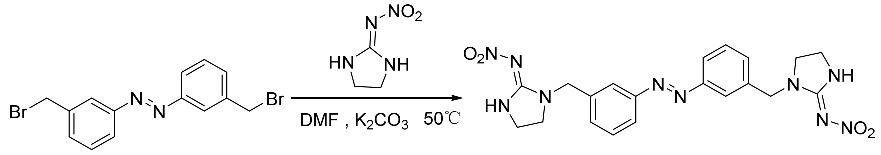

Amino-2-nitromethylene-imidazolidine (0.260 g, 2.0 mmol) and K_2CO_3 (0.414 g, 3.0 mmol) in DMF (15 mL) was stirred at 50°C. Then, compound **10a** (0.367 g, 1 mmol) in DMF was added. The mixture was stirred at 50 °C for 24 h and the progress of the reaction was monitored by TLC analysis. After completion, the reaction was evaporated under reduced pressure to remove DMF, diluted with water and extracted with dichloromethane (10 mL × 6). The combined organic layers were dried over Na_2SO_4 and concentrated. The crude product was further purified by column chromatography using dichloromethane and acetone (v:v = 15:1) as eluent. Orange solid, yield 19.8 %, m.p. = 232.3-233.3 °C; ^{1}H NMR (400 MHz, DMSO-d_6): δ 9.01 (s, 1H), 7.85 (d, J = 8.0 Hz, 1H), 7.82 (s, 1H), 7.62 (m, 1H), 7.51 (d, J = 7.6 Hz, 1H), 4.59 (s, 2H), 3.66 (t, J = 9.0 Hz, 2H), 3.51 (t, J = 9.0 Hz, 2H); ^{13}C NMR (100 MHz, DMSO-d_6): δ 160.81, 149.43, 139.47, 136.56, 129.91, 124.22, 122.17, 46.56, 45.59, 45.33; HRMS (ESI): m/z calcd for $C_{20}H_{22}N_{10}O_4Na$ $[M+Na]^+$ 489.1723, found 489.1737.

*(E)-1-(4-(((E)-2-(nitromethylene)imidazolidin-1-yl)methyl)phenyl)-2-(4-(((Z)-2-(nitromethylene)imidazolidin-1-yl)methyl)phenyl)diazene (**AMI-8**)*

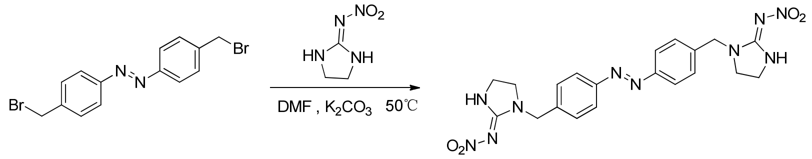

Amino-2-nitromethylene-imidazolidine (0.260 g, 2.0 mmol) and K_2CO_3 (0.414 g, 3.0 mmol) in DMF (15 mL) was stirred at 50°C. Then, compound **10b** (0.367 g, 1 mmol) in DMF was added. The mixture was stirred at 50°C for 24 h and the progress of the reaction was monitored by TLC analysis. After completion, the reaction was evaporated under reduced pressure to remove DMF, diluted with water and extracted with dichloromethane (10 mL × 6). The combined organic layers were dried over Na_2SO_4 and concentrated. The crude product was further purified by column chromatography using dichloromethane and acetone (v:v = 15:1) as eluent. Orange solid, yield 11.0 %, m.p. = 271.2-272.0 °C; ^{1}H NMR (400 MHz, DMSO-d_6): δ 9.00 (s, 2H), 7.91 (d, J = 8.0 Hz, 2H), 7.51 (d, J = 8.0 Hz, 2H), 4.56 (s, 4H), 7.57-7.46 (m, 4H), 3.69-3.65 (m, 4H), 3.54-3.49 (m, 2H); ^{13}C NMR (100 MHz, DMSO-d_6): δ 160.83, 129.16, 127.59, 123.38, 122.94, 47.71, 45.52, 42.05; HRMS (ESI): m/z calcd for $C_{20}H_{22}N_{10}O_4Na$ $[M+Na]^+$ 489.1723, found 489.1732.

*(E)-1-(3-(((E)-3-((6-chloropyridin-3-yl)methyl)-2-(nitromethylene)imidazolidin-1-yl)methyl)phenyl)-2-(3-(((Z)-3-((6-chloropyridin-3-yl)methyl)-2-(nitromethylene)imidazolidin-1-yl)methyl)phenyl)diazene (**AMI-9**)*

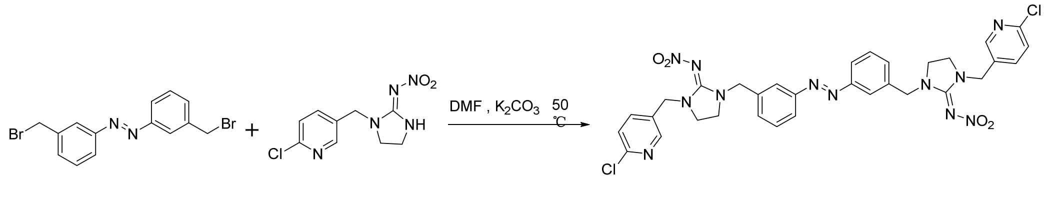

Imidacloprid (0.765 g, 3.0 mmol) and K_2CO_3 (0.58 g, 4.0 mmol) in DMF (2 mL) stirred at 50°C. Then, compound **10a** (0.55 g, 1.5 mmol) in DMF was added. The mixture was kept stirring at 50°C for 24 h and the progress of the reaction was monitored by TLC analysis. After completion, the reaction was evaporated under reduced pressure to remove DMF, diluted with water and extracted with dichloromethane (10 mL × 6). The combined organic layers were dried over Na_2SO_4 and concentrated. The crude product was further purified by column chromatography using dichloromethane and acetone (v:v = 4:1) as eluent. Orange solid, yield 26.7 %, m.p. 200.6-201.5 °C; ^{1}H NMR (400 MHz, DMSO-d_6) δ 8.40 (s, 1H), 7.86 (m, 3H), 7.64 (m, 1H), 7.55 (m, 2H), 4.58 (s, 2H), 4.50 (s, 2H), 3.69 (s, 4H); ^{13}C NMR (101 MHz, DMSO) δ 161.30, 152.59, 152.52, 150.20, 149.92, 139.98, 137.07, 130.93, 130.40, 124.76, 122.72, 122.48, 49.66, 47.06, 46.09, 45.86; HRMS (ESI+): m/z calcd for $C_{32}H_{31}N_{12}O_4{}^{35}Cl_2$ $(M+H)^+$, 717.1968; found 717.1954; calcd for $C_{32}H_{31}N_{12}O_4{}^{35}Cl^{37}Cl$ $(M+H)^+$, 719.1939; found, 719.1936; calcd for $C_{32}H_{31}N_{12}O_4{}^{37}Cl_2$ $(M+H)^+$, 721.1909; found, 721.1928.

(E)-1-(4-(((E)-3-((6-chloropyridin-3-yl)methyl)-2-(nitromethylene)imidazolidin-1-yl)methyl)phenyl)-2-(4-(((Z)-3-((6-chloropyridin-3-yl)methyl)-2-(nitromethylene)imidazolidin-1-yl)methyl)phenyl)diazene ***(AMI-10)***

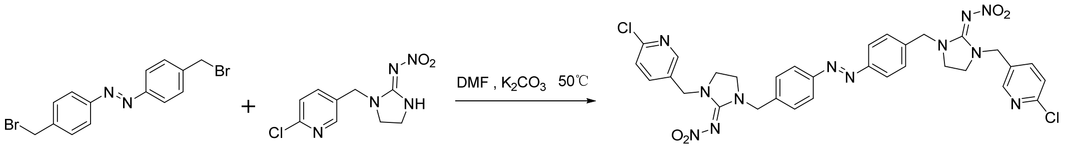

Imidacloprid (0.765 g, 3.0 mmol) and K_2CO_3 (0.58 g, 4.0 mmol) in DMF (2 mL) stirred at 50°C. Then, compound **10b** (0.55 g, 1.5 mmol) in DMF was added. The mixture was kept stirring at 50°C for 24 h and the progress of the reaction was monitored by TLC analysis. After completion, the reaction was evaporated under reduced pressure to remove DMF, diluted with water and extracted with dichloromethane (10 mL × 6). The combined organic layers were dried over Na_2SO_4 and concentrated. The crude product was further purified by column chromatography using dichloromethane and acetone (v:v = 4:1) as eluent. Orange solid, yield 15.3 %, m.p. 213.8-214.5 °C; ^{1}H NMR (400 MHz, DMSO-d_6): δ 8.40 (d, J = 2.0 Hz, 1H), 7.92 (d, J = 8.0 Hz, 4H), 7.84 (dd, J_1 = 2.4 Hz, J_2 = 8.0 Hz, 2H), 7.58 (d, J = 8.4 Hz, 2H), 7.55 (d, J = 8.4 Hz, 4H), 4.56 (s, 4H), 4.51 (s, 4H), 3.70 (s, 8H) ppm; ^{13}C NMR (100 MHz, DMSO-d_6) δ 161.33, 151.96, 150.21, 149.94, 139.99, 139.11, 130.94, 129.45, 124.79, 123.36, 49.63, 47.05, 46.09, 45.92 ppm; HRMS (ESI): m/z calcd for $C_{32}H_{30}N_{12}O_4{}^{35}Cl_2Na$ $[M+Na]^+$ 739.1788, found 739.1788; calcd for $C_{32}H_{30}N_{12}O_4{}^{35}Cl^{37}ClNa$ $[M+Na]^+$ 741.1758, found 741.1770; calcd for $C_{32}H_{30}N_{12}O_4{}^{37}Cl_2Na$ $[M+Na]^+$ 743.129, found 743.1748.

2. Photochromism

The UV-Vis spectrums upon irradiation were depicted in Figure S3.

The photostationary states were analyzed by HPLC measurements, exemplified by **AMI-10** in Figure S4.

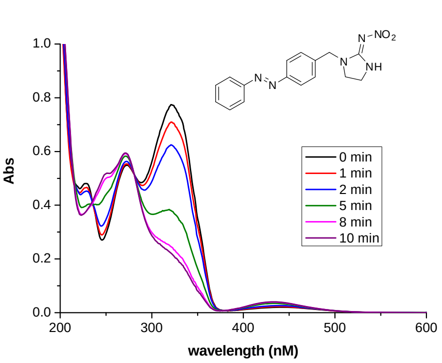
Abs
wavelength (nM)
0 min
1 min
2 min
5 min
8 min
10 min

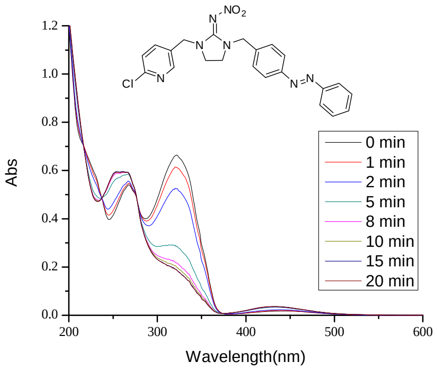
Abs
Wavelength(nm)
0 min
1 min
2 min
5 min
8 min
10 min
15 min
20 min

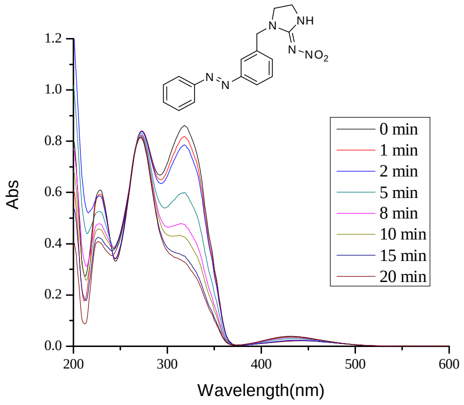
Abs
Wavelength(nm)
0 min
1 min
2 min
5 min
8 min
10 min
15 min
20 min

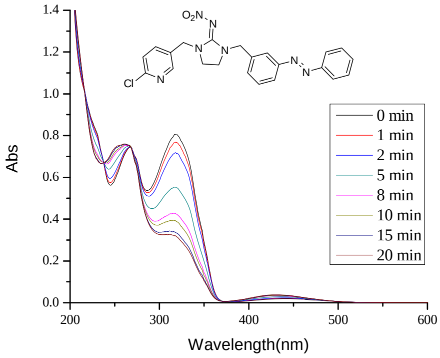
Abs
Wavelength(nm)
0 min
1 min
2 min
5 min
8 min
10 min
15 min
20 min

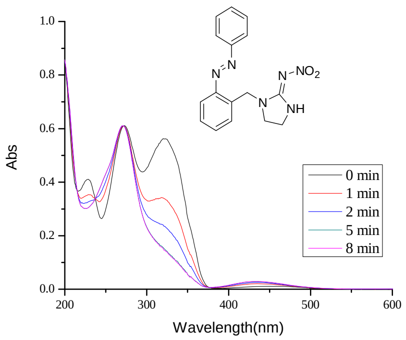
Abs
Wavelength(nm)
0 min
1 min
2 min
5 min
8 min

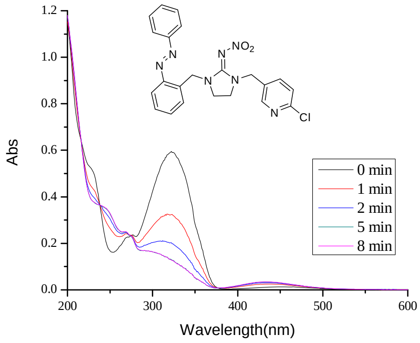
Abs
Wavelength(nm)
0 min
1 min
2 min
5 min
8 min

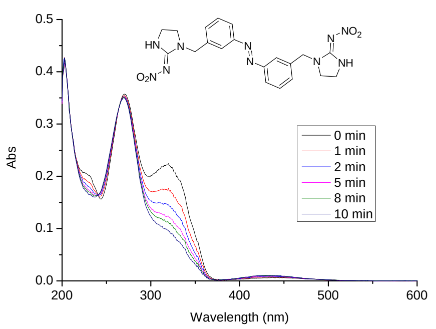
Abs
Wavelength (nm)
0 min
1 min
2 min
5 min
8 min
10 min

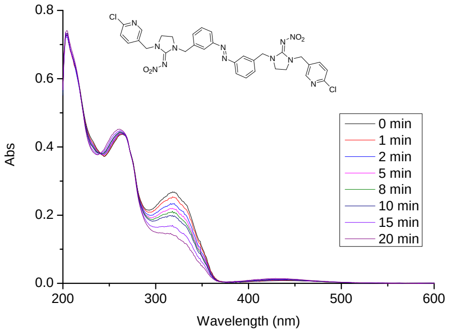
Abs
Wavelength (nm)
0 min
1 min
2 min
5 min
8 min
10 min
15 min
20 min

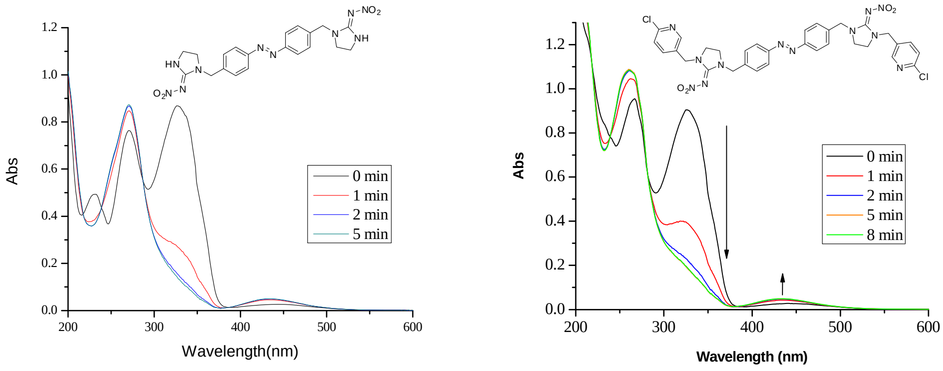

Supplementary Fig. S3. UV-Vis spectrums upon irradiation

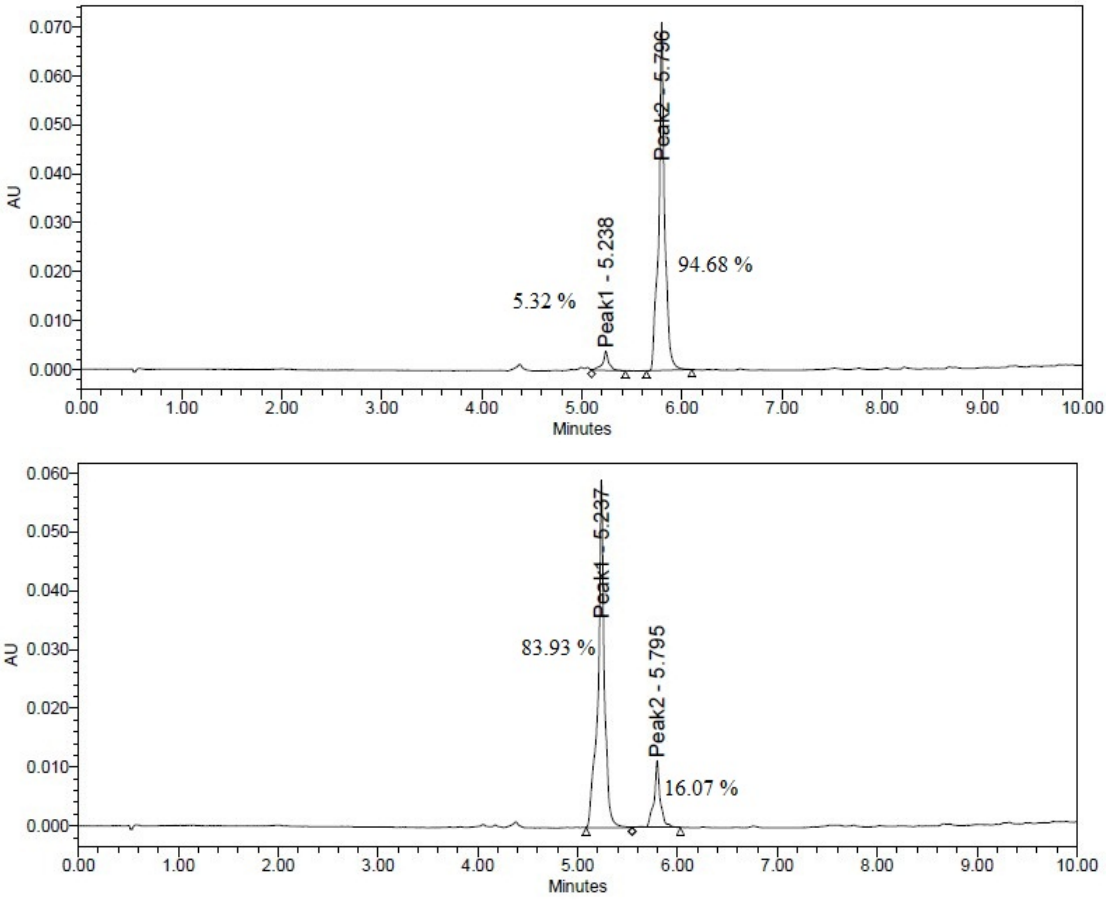

Supplementary Fig. S4. The ratio changes of **AMI-10** upon irradiation determined by HPLC
